# Implantable Cardiac Devices in Patients with Brady- and Tachy-Arrhythmias: An Update of the Literature

**DOI:** 10.31083/j.rcm2505162

**Published:** 2024-05-11

**Authors:** William Chick, Christopher Monkhouse, Amal Muthumala, Syed Ahsan, Nikolaos Papageorgiou

**Affiliations:** ^1^Cardiology Department, Lister Hospital, East and North NHS Hertfordshire NHS Trust, SG1 4AB Stevenage, UK; ^2^Electrophysiology Department, Barts Heart Centre, St. Bartholomew's Hospital London, Barts Health NHS Trust, EC1A 7BE London, UK; ^3^Institute of Cardiovascular Science, University College London, WC1E 6BT London, UK

**Keywords:** pacemaker, implantable cardioverter-defibrillator, cardiac resynchronisation therapy, tachy-arrhythmia, brady-arrhythmia

## Abstract

Implantable cardiac devices are a vital treatment option in the management of tachy/brady-arrhythmias and heart failure with conduction disease. In the recent years, these devices have become increasingly sophisticated, with high implantation success rates and longevity. However, these devices are not without risks and complications, which need to be carefully considered before implantation. In an era of rapidly evolving cardiac device therapies, this review article will provide an update on the literature and outline some of the emerging technologies that aim to maximise the efficiency of implantable devices and reduce complications. We discuss novel pacing techniques, including alternative pacing sites in anti-bradycardia and biventricular pacing, as well as the latest evidence surrounding leadless device technologies and patient selection for implantable device therapies.

## 1. Introduction 

Implantable cardiac devices are at the forefront of managing tachy-arrhythmias, brady-arrhythmias and heart failure with concomitant conduction disease. They reduce mortality and morbidity in selected patients and provide improvements in quality of life and functional status of patients. However, these devices are not without risks. Complications can occur at the time of implantation including bleeding, infection and damage to surrounding myocardial structures, as well as later on, including lead failure, dislodgement or pacemaker induced cardiomyopathy. Recent advances in cardiac devices aim to reduce some of these risks through more physiological pacing techniques or leadless designs. In this review article we provide an update on the literature on implantable cardiac devices, focusing on new pacing techniques, leadless devices and patient selection for device therapies.

## 2. Pacemakers for Brady-Arrhythmias

**Fig. 1.**
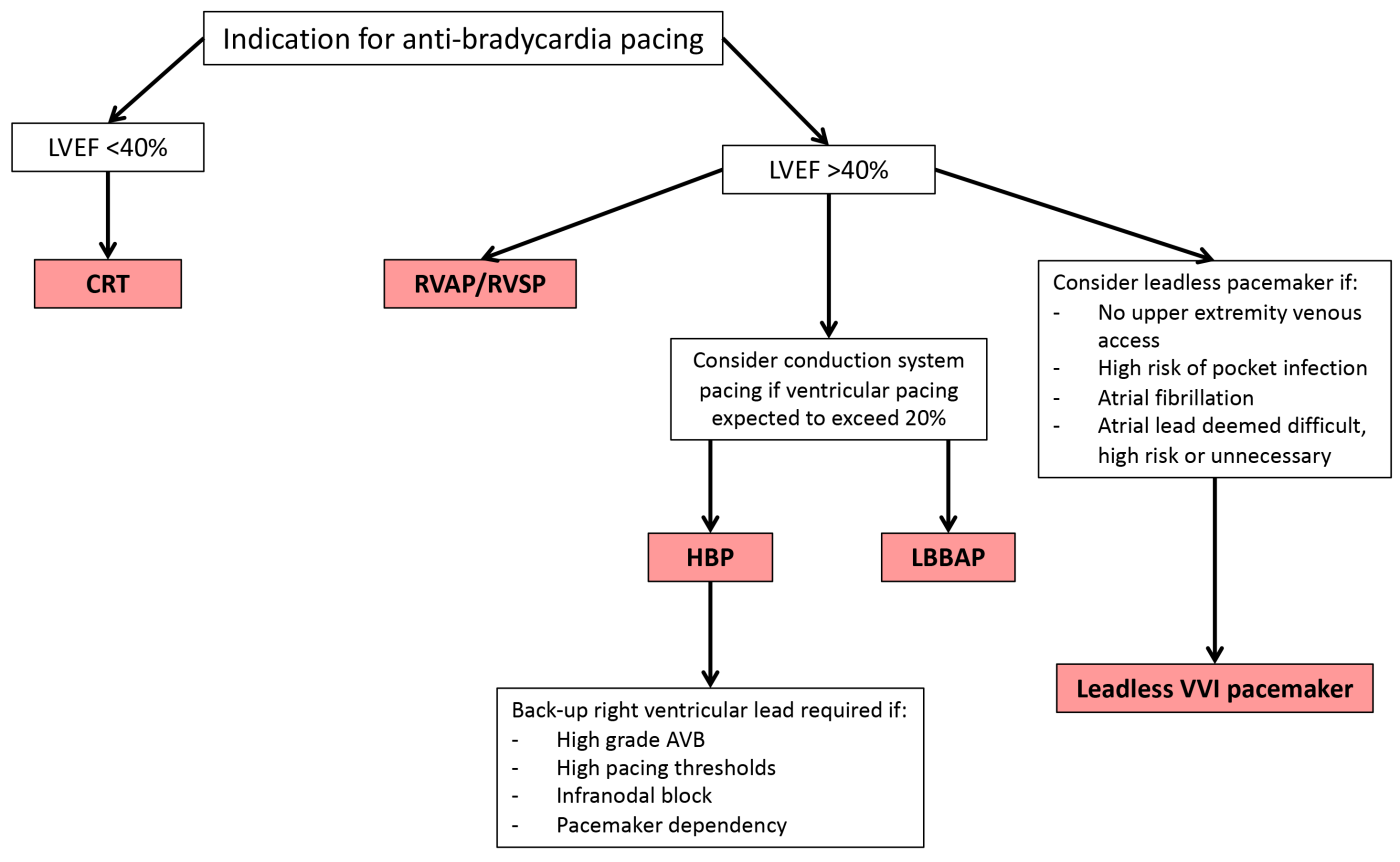
**Flow diagram to aid the selection of device in the management of brady-arrhythmias**. LVEF, left ventricular ejection fraction; CRT, cardiac resynchronisation therapy; RVAP, right ventricular apical pacing; RVSP, right ventricular septal pacing; HBP, his-bundle pacing; LBBAP, left bundle branch area pacing; AVB, atrioventricular block.

Right ventricular apical pacing (RVAP) is the most common ventricular pacing site. However, it can be associated with ventricular desynchrony, pacing induced cardiomyopathy and impaired ventricular function [1]. In turn, pacing-induced cardiomyopathy results in significant morbidity and mortality, with higher rates of atrial fibrillation (AF), heart failure hospitalisation and cardiovascular mortality [2, 3]. As a result of this, several alternative pacing sites have been proposed for both single and dual chamber pacemakers, including the right ventricular septum (RVS) and conduction system (His-bundle (HB) and left bundle branch area (LBBA)). These novel pacing sites, along with the development of leadless pacemakers, mean clinicians have several treatments options in the management of brady-arrhythmias (Fig. 1).

### 2.1 Right Ventricular Septal Pacing

Randomised controlled trials and retrospective observational studies have identified reduced ventricular desynchrony and left ventricular (LV) strain in patients with right ventricular (RV) mid or high septal pacing [4, 5, 6]. Whether this necessarily translates to better LV function and improved clinical outcomes is uncertain. A meta-analysis in 2015 compared non-apical RV pacing and RVAP in patients with preceding LV impairment and found RVAP was associated with a significantly greater deterioration in left ventricular ejection fraction (LVEF) [7]. The analysis, however, had significant heterogeneity and included a range of non-apical pacing sites including the RV outflow tract, RVS and HB. More recently a meta-analysis by Zhuang *et al*. [8] found that RVS pacing was associated with better LV function compared to RVAP in studies with a follow up of less than 12 months. This was not, however, observed in studies with longer follow-up periods. The authors also compared His-bundle pacing (HBP) to RVAP and although there was a trend towards better LV function in the HBP group, given there were only three small trials included in the analysis, this did not achieve statistical significance. Since this meta-analysis was published, two large retrospective observational studies have been published on the matter reporting no differences in pacemaker induced cardiomyopathy, heart failure hospitalisations or all-cause mortality between patients receiving RVAP and RVS pacing [9, 10]. Moreover, the results of the SEPTAL-PM trial found no difference in LVEF, quality of life scores and functional status between RVS pacing and RVAP at 18 months [11]. Only one study has reported a higher risk of heart failure events with RVAP, however this study included high numbers of patients with co-morbidities known to be associated with heart failure events, such as baseline LV impairment and AF, when compared to other studies [12]. As a result of this, current guidelines do not recommend the preferential use of either RVAP or RVS pacing over the other.

### 2.2 Conduction System Pacing

In a 2022 meta-analysis comparing HBP to right ventricular pacing (RVP), Abdin *et al*. [13] found that HBP was associated with a lower risk of heart failure hospitalisation, new AF and a reduction in LVEF when compared to RVP. There was a trend towards reduced all-cause mortality in the HBP group, but this was not statistically significant. They noted that HBP was associated with significantly increased procedure and fluoroscopy times, as well as increased need for lead revision. Lead revision was mostly due to progressive increases in His-capture thresholds. The HB is anatomically short, making pacing difficult and often right sided chamber back-up electrodes are required due to high rates of electrode displacements [14]. As a result, European Society of Cardiology (ESC) guidelines suggest RV leads should be considered in patients with high capture thresholds, atrioventricular node ablation or in patients with either high-degree or infranodal block [15]. As well as this, issues with high capture thresholds and losing HB capture over time are well documented in the literature and remain a major issue with this form of pacing.

An emerging alternative to HBP is left bundle branch area pacing (LBBAP). In LBBAP, a pacing lead is screwed into the LV septum, allowing pacing more distally along the intrinsic conduction system. It is associated with high success rates, likely due to a combination of deep fixation and the wide target area covered by the LBBA [16, 17]. Studies have reported low and stable capture thresholds, which in turn leads to battery longevity and a reduced need for generator changes [18].

As well as displaying electrocardiographic and echocardiographic benefits over RVAP, there are now several observational studies suggesting it is associated with better clinical outcomes [17, 19, 20]. Sharma *et al*. [21] showed that RVP was associated with a significantly higher risk of all-cause mortality and heart failure hospitalisation when compared to LBBAP. Interestingly when they analysed patients with high pacing burdens (>40%), the RVP cohort had significantly higher mortality, heart failure hospitalisations and need for upgrading to biventricular pacing (BiVP), however there was no difference between the groups with regards to these outcomes in patients with low pacing burdens (<20%). Similarly, Chen *et al*. [22] found that LBBAP was associated with a lower risk of all-cause mortality, heart failure hospitalisation and unexplained syncope when compared to RVP. After propensity matching, they found reduced ventricular desynchrony and LV strain in the LBBAP cohort, but with no significant differences in LVEF between the groups. Importantly, these studies report similar numbers of lead failures when compared to RVP, therefore suggesting it is a safe and viable alternative to RVP.

There is a need for more randomised controlled trials with long follow-up periods both comparing conduction system pacing (CSP) to RVP but also comparing HBP to LBBAP. As a result, the ESC has avoided formulating recommendations for LBBAP at this time, although they concede this is likely to change in the future [15].

### 2.3 Leadless Pacemakers 

Leadless pacemakers are delivered using femoral venous access and are implanted directly into the RV wall, providing single chamber pacing. They offer an alternative to transvenous systems in patients with poor venous access, such as subclavian or superior vena cava occlusion, recurrent pacemaker infections or indwelling central catheters such as end stage renal patients.

The safety profile of leadless pacemakers has been extensively researched, and there are several meta-analyses comparing the safety profile of leadless and transvenous systems [23, 24]. In their analyses of 17 studies, Shtembari *et al*. [25] found leadless pacemakers may be safer than transvenous systems, with significantly lower risks of complications, more specifically device dislodgement and pneumothoraces. They did however report 2.65 times higher odds of pericardial effusions. A further meta-analysis found reduced rates of endocarditis in patients with leadless devices, and a trend towards reduced haematomas and haemothoraces post-implantation, however this did not achieve statistical significance [26]. Unlike transvenous devices that have been shown to double the risk of significant tricuspid regurgitation [27], Haeberlin *et al*. [28] found that leadless devices are not associated with worsening tricuspid regurgitation . Shtembari *et al*. [25] found that patients receiving leadless devices had 46% lower odds of re-intervention compared to transvenous devices, which is likely explained by the historical technical difficulty of changing or retrieving these devices, as well as the careful selection of leadless device candidates. Despite studies reporting device retrieval success rates of 85–94%, it remains a technically difficult procedure with potential risks such as arteriovenous fistulas and pulmonary artery embolization [29, 30, 31]. Due to both this, and the lack of long-term data on performance, ESC guidelines suggest there should be careful selection of patients suitable for these devices, especially in younger cohorts [15].

A large observational study by El-Chami *et al*. [32] found no difference in adjusted 2-year all-cause mortality between patients with leadless or transvenous pacemakers. However, before adjustment for clinical characteristics, a higher mortality rate was observed in the leadless arm of the study. Similarly, Bodin *et al*. [33] found mortality rates were higher in patients receiving leadless devices but after propensity matching there was no significant difference in all-cause mortality and cardiovascular related mortality. Garg *et al*. [34] compared outcomes between patients receiving transvenous pacemakers, and patients receiving leadless devices due to them being unsuitable for a transvenous device, and found mortality was higher in the leadless device group. There was, however, a significantly higher rate of co-morbidities in the group receiving leadless devices including diabetes, end-stage renal disease and previous failed transvenous devices. The high burden of co-morbidities in patients receiving leadless pacemakers likely also explains the higher mortality rates reported in the El-Chami *et al*. [32] and Bodin *et al*. [33] studies, prior to them adjusting for these variables.

Interestingly, in patients undergoing pace and ablate strategies, Sanchez *et al*. [35] found a reduced rate of pacemaker induced cardiomyopathy in patients receiving leadless devices versus transvenous pacemakers. Most leadless devices in this study were implanted in the mid-septum location, which as covered elsewhere may be associated with reduced ventricular desynchrony. As well as a favourable safety profile and potential reduction in pacemaker induced cardiomyopathy, evidence also suggests that leadless devices are associated with improved quality of life outcomes. Cabanas-Grandío *et al*. [36] found leadless devices were well tolerated, less painful and associated with better physical function and mental health at 6 months follow-up.

However, leadless ventricular pacemakers only provide single chamber pacing, thereby limiting their use to only 20% of patients that require permanent pacing [37]. Therefore they are only indicated in patients with AF, at high risk of infection, with poor venous access or in cases where an atrial lead is deemed difficult, high risk or unnecessary for effective therapy [15, 38]. To expand their use to dual-chamber pacing, there needs to be bidirectional communication between more than one leadless device. Cantillon *et al*. [39] proved this was possible in ovine subjects with the addition of a leadless device in the right atrium, thereby enabling atrioventricular synchrony. In an international, multicentre analysis, Knops *et al*. [40] has now demonstrated the efficacy of this technology in humans. They reported a 98.3% implantation success rate with high rates of atrioventricular synchrony. After 90 days, only 9.7% of patients developed device or implantation complications, which was mostly the development of AF. Atrial device dislodgement occurred in 3.4% of patients but all were successfully retrieved.

Despite an ever-growing evidence base outlining the safety and efficacy of leadless pacemakers, the technology remains in the early stages of development, and this is reflected in clinical guidelines. It should be noted that all studies analysing outcomes of leadless pacemakers to date have been observational studies, and there are currently no randomised controlled trials comparing outcomes of leadless and conventional, transvenous devices.

## 3. Cardiac Re-Synchronisation Therapy 

Cardiac resynchronisation therapy (CRT) aims to reduce the degree of electromechanical desynchrony in patients with heart failure and conduction disease by coordinating contractions between the ventricles, thereby resulting in reverse ventricular remodelling and improvements in cardiac function. It has been shown to improve the functional status of patients, reduce heart failure admissions and reduce mortality in patients with heart failure with a reduced ejection fraction (HFrEF) and concomitant conduction tissue disease [41, 42, 43]. Results from large scale studies including the COMPANION, CARE-HF and PATH-CHF trials support its use in patients with New York Heart Association (NYHA) class III to IV [44, 45, 46].

Despite the success of CRT, one of the major issues associated with these devices is the large number of patients that fail to respond to the therapy. Although there is new evidence to suggest that stabilisation of cardiac function should also be considered a successful outcome of CRT [47], around a third of patients with CRT are considered “non-responders” due to no improvements in LV function after device implantation [48]. This therefore results in unnecessary costs, hospitalisations, and invasive procedures. Reasons for non-response include unfavourable anatomy, including coronary sinus stenosis, venous malformations or close proximity to the phrenic nerve, as well as lead related complications including lead displacement or suboptimal pacing thresholds [49].

### 3.1 Conduction System CRT

As discussed already, CSP involves pacing part of the native ventricular conduction system thereby allowing physiological depolarisation and narrowing the QRS interval [50]. Reductions in the QRS interval are associated with reductions in mortality, as well as higher chances of echocardiographic response in patients undergoing BiVP [51, 52, 53]. There are several alternative pacing sites that have been suggested, most notably the HB and LBBA. In observational studies comparing HBP, LBBAP and BiVP, CSP strategies have been associated with favourable outcomes with regards to LV function, QRS duration and functional status [54, 55, 56]. In a recent meta-analysis, CSP has been shown to not only shorten QRS intervals and improve LV function when compared to BiVP, but also increase the odds of patients being echocardiographic responders or super-responders [57]. This has also been translated into improved clinical outcomes, with 63% lower odds of heart failure hospitalisation and 39% lower odds of mortality in patients receiving CSP-CRT.

### 3.2 His-Bundle CRT

Deshmukh *et al*. [58] were the first to show the efficacy of permanent HBP in CRT, and since then it has gone from being considered a bail-out option when LV lead placements are unsuccessful, to being widely considered as a primary alternative to BiVP. Despite offering physiological pacing, it is often complicated by high and sometimes unstable correction thresholds [59].

In their study, Huang *et al*. [60] found HB capture was achieved in 100% of patients and corrected left bundle branch block (LBBB) in 97.3%. In this analysis, only 75.7% went on to receive permanent HBP, due to either failure of lead fixation or high correction thresholds. Patients with HBP demonstrated significant improvements in LV function, NYHA classification, B-type natriuretic peptide (BNP) values and a variety of echocardiographic measures. Significantly, 89% of patients achieved a greater than 50% improvement in LVEF after one year. A multi-centre study by Sharma *et al*. [61] also demonstrated the safety and clinical efficacy of permanent HBP. The majority of the patients in their analysis were patients receiving HBP as a primary pacing strategy, but they also included non-responders to BiVP CRT and patients with LV lead implantation failure. HBP resulted in statistically significant improvements in QRS duration, LVEF and NYHA classifications, and results were comparable with those receiving BiVP CRT. Despite these positive findings, in 7% of cases there was an increase in HB capture threshold, and 3% of patients lost left bundle branch recruitment late in the 14-month follow up period.

Promising findings from observational analyses has led to the first randomised controlled trials comparing HBP to conventional BiVP. In the His-SYNC trial, unlike BiVP, HBP resulted in significant reductions in the QRS interval [62]. Furthermore, HBP achieved a slightly greater improvement in LVEF compared to BiVP, however this did not achieve statistical significance. It should be noted that there was significant crossover during the trial, with results analysed using an intention-to-treat approach. In the largest randomised controlled trial comparing HBP to BiVP to date, Vinther *et al*. [63] found HBP was successful in 72% of patients, with the remaining patients crossing over to the BiVP arm due to high capture thresholds or an inability to detect the His signal. Procedural data showed HBP patients had longer procedural times, but after excluding patients that crossed arms of the study, there were comparable procedure times, fluoroscopy and X-ray dosages. At 6 months follow-up patients who had undergone HBP had significantly higher LVEF than the BiVP group, however there were no significant differences in the change in LVEF between baseline and 6 months between the groups. Both groups demonstrated similar significant improvements in QRS duration, BNP levels, performance status and echocardiographic parameters.

The findings from observational and randomised controlled trials suggest HBP is a safe and effective alternative to BiVP in appropriate patients. However, many of the trials comparing the outcomes of HBP and alternative pacing strategies are non-randomised observational studies or randomised controlled trials with small cohorts. A recent meta-analysis pooled the results of these studies comparing HBP to BiVP and found the former resulted in significantly shorter QRS intervals, higher LVEF’s and increased odds of patients being an echocardiographic responder or super-responder to CRT [57]. However, this did not translate to clinical outcomes, because although HBP was associated with a trend towards reduced mortality and hospitalisation when compared to BiVP, it did not achieve statistical significance. This highlights the need for further, high-powered randomised controlled trials comparing the outcomes of HBP with other CRT pacing modalities.

### 3.3 Left Bundle-Branch Area CRT

An alternative to HBP is through pacing the LBBA. By pacing more distally to the conduction block, studies have shown that when compared to HPB, LBBAP may be associated with lower and more stable thresholds and a higher likelihood of correcting LBBB [64, 65]. Furthermore, lead implantation and capture may be easier than coronary sinus LV leads. The left conduction system has a wide network and is easily captured by screwing a lead into the LV septum endomyocardium, whereas coronary sinus anatomy varies greatly between patients meaning it can be difficult to place the lead in the optimal venous branch. LBBAP was first described in a case report in 2017, and since then there have been several observational studies demonstrating favourable outcomes in patients undergoing LBBAP versus BiVP [66, 67, 68, 69, 70, 71, 72, 73] (Table 1).

**Table 1.** **Studies comparing left bundle branch area cardiac resynchronisation therapy (LBBA-CRT) to biventricular cardiac resynchronisation therapy (BiV-CRT)**.

Study	Study design	Operative time	Country	Follow-up	Patients (n)	QRS interval at follow-up	NYHA class at follow-up	LVEF at follow-up	Echocardiographic response	Clinical outcomes
Guo *et al*. 2020 [66]	Prospective propensity matched observational study	2018–2019	China (single centre)	Mean 14.3 ± 7.2 months	42 (21 BiV-CRT, 21 LBBA-CRT)	Greater reduction in QRS duration in the LBBA-CRT group (56.0 ± 14.7 ms vs 32.3 ± 14.6 ms, *p * < 0.0001)	Trend towards lower NYHA class in LBBA-CRT group but not significant (1.3 ± 0.9 vs 1.5 ± 0.7, *p* = 0.06)	Both groups reported significant improvements in LVEF. No significant difference between the groups but trend towards better LVEF in LBBA-CRT group (50.9 ± 10.7% vs 44.4 ± 13.3%, *p* = 0.12)	Greater response (90.5% vs 80.9%, *p* = 0.43) and super response (80.9% vs 57.1%, *p* = 0.09) in the LBBA-CRT group compared to the BiV-CRT group but not statistically significant	No heart failure hospitalization, ventricular arrhythmias or mortality reported in either group
Li *et al*. 2020 [67]	Prospective propensity matched observational study	2018	China (multi-centre)	6 months	81 (54 BiV-CRT, 27 LBBA-CRT)	Greater reduction in QRS duration in LBBA-CRT group (58.0 ms vs 12.5 ms, *p * < 0.001)	Lower NYHA class in LBBA-CRT group (1.5 ± 0.5 vs 2.3 ± 0.7, *p * < 0.001)	Greater improvement in LVEF in LBBA-CRT group (17.1% vs 7%, *p * < 0.001)	Greater response (88.9% vs 66.7%, *p* = 0.035) and super-response (44.4% vs 16.7%, *p* = 0.007) in LBBA-CRT group	-
Wang *et al*. 2020 [68]	Prospective propensity matched observational study	-	-	6 months	40 (10 LBBA-CRT, 30 BiV-CRT)	Greater reduction in QRS duration in LBBA-CRT group (60.80 ± 20.09 ms vs 33.00 ± 21.48 ms, *p* = 0.0009)	Greater percentage of patients grade I-II in LBBA-CRT group (median 1.5 vs 2.0, *p* = 0.029)	LVEF 45.7 ± 9.2% in LBBA-CRT group and 39.3 ± 12.3% in BiV-CRT group at follow-up	Greater response rate in LBBA-CRT group (100% vs 63.3%, *p* = 0.038)	No mortality in either group, one heart failure hospitalisation in the BiV-CRT group
Liu *et al*. 2021 [69]	Prospective observational study	2018–2021	China (multi-centre)	Mean 4.0 ± 1.4 months	62 (35 BiV-CRT, 27 LBBA-CRT)	Greater reduction in QRS duration in LBBA-CRT group (64.1 ± 18.9 ms vs 32.5 ± 22.3 ms, *p * < 0.001)	Greater improvement of NYHA class in LBBA-CRT group (−1.6 ± 0.6 vs −0.9 ± 0.8, *p* = 0.001)	Greater improvement in LBBA-CRT group but not statistically significant (17.2 ± 9.3 vs 13.7 ± 11.5, *p* = 0.113)	Greater response rate in LBBA-CRT group (88.9% vs 68.6%)	-
Hua *et al*. 2022 [70]	Prospective observational study	2018–2019	China (single centre)	Mean 23.7 ± 4.4 months	41 (20 BiV-CRT, 21 LBBA-CRT)	Greater reduction in QRS duration in LBBA-CRT group (48.6 ± 26.29 ms vs 20.7 ± 28.3 ms, *p* = 0.002)	Greater improvement of NYHA class in LBBA-CRT group but not statistically significant (–1.2 ± 0.9 vs 1.1 ± 0.7, *p* = 0.75)	Greater improvement in LBBA-CRT group but not statistically significant (15.7 ± 14.6% vs 12.8 ± 11.1%, *p* = 0.509)	Greater super-response rate in LBBA-CRT group but not statistically significant (42.9% vs 35.0%, *p* = 0.606)	Lower number of hospitalisations in LBBA-CRT group (*p* = 0.019). No difference in mortality between the groups
Chen *et al*. 2022 [71]	Prospective observational study	2018–2019	China (multi-centre)	12 months	100 (51 BiV-CRT, 49 LBBA-CRT)	Greater reduction in QRS duration in LBBA-CRT group versus BiV-CRT group (59.2 ± 16 ms vs 31 ± 11.3 ms, *p * < 0.001)	Both groups had significant improvements in NYHA class, but greater number of patients class III-IV in the BiV-CRT group (19.6% vs 4.1%, *p* = 0.028)	Greater improvement in LBBA-CRT group (20.9 ± 11.8% vs 15.2 ± 10%, *p* = 0.015)	Greater response (85.71% vs 80.39%, *p* = 0.479) and super-response rate (61.2% vs 39.2%, *p * < 0.001) in LBBA-CRT group	Heart failure hospitalisations in two LBBA-CRT patients and five BiV-CRT patients. No mortality in either group
Wang *et al*. 2022 [72]	Randomised controlled trial	2019–2021	China (multi-centre)	6 months	40 (20 BiV-CRT, 20 LBBA-CRT)	Greater reduction in QRS duration in LBBA-CRT group (45.4 ms vs 36.2 ms) but not statistically significant	Greater reduction in NYHA class in LBBA-CRT group (–1.2 ± 0.1 vs –1.1 ± 0.1) but not statistically significant	Greater improvement in LBBA-CRT group (21.1% vs 15.6%, *p* = 0.039)	Greater number of super-responders in LBBA-CRT group (65% vs 42.1%)	No heart failure admissions, ventricular arrhythmias or mortality in both groups
Diaz *et al*. 2023 [73]	Prospective observational study	2020–2022	United States of America, Colombia, Argentina (multi-centre)	Median 340 days	371 (243 BiV-CRT, 128 LBBA-CRT)	Shorter QRS durations in the LBBA-CRT group (123.7 ± 18.8 ms vs 149.3 ± 29.1, *p * < 0.001)	Greater percentage of patients improving by at least one NYHA class (80.4% vs 67.9%, *p * < 0.001)	Greater improvement in LBBA-CRT group (8% ± 9.9% vs 3.9% ± 7.9%, *p * < 0.001)	-	Reduced heart failure hospitalisations in LBBA-CRT group (22.6% vs 39.5% *p* = 0.021), trend towards reduced mortality in LBBA-CRT group but not significant (5.5% vs 11.9%, *p* = 0.19)

BiV-CRT, biventricular cardiac resynchronisation therapy; LBBA-CRT, left bundle branch area cardiac resynchronisation therapy; NYHA, New York Heart Association; LVEF, left ventricular ejection fraction.

Large observational studies comparing LBBAP to BiVP have suggested that, as well as significantly reducing QRS intervals, LBBAP is associated with greater improvements in LV function and higher chances of echocardiographic response [71, 73]. Diaz *et al*. [73] found that this also translated to better clinical outcomes, with reductions in heart failure hospitalisations and a trend towards reduced mortality. Despite shorter procedural and fluoroscopy times compared to BiVP, LBBAP did have lower rates of left bundle branch capture, which may be explained by a lack of operator experience especially in earlier cases. They found no significant differences between the groups with regards to acute or long-term device related complications, including lead displacement. The only randomised controlled trial comparing LBBAP to BiVP to date, also found that LBBAP resulted in greater improvements in LV function [72]. Both groups reported improvements in BNP levels, QRS duration and NYHA functional class however there were no statistically significant differences between them. Moreover, there were no re-admissions or deaths in either treatment arms at 6 months. It should be noted, however, that this study only included patients with non-ischaemic cardiomyopathy (NICM).

Success rates of LBBAP CRT are around 82–84% [73, 74]. However, there appears to be a steep learning curve with the majority of failures occurring in inexperienced operators [75, 76]. Furthermore, there is evidence to show that as operators become more experienced with LBBAP, not only do success rates increase, but fluoroscopy times reduce and QRS durations shorten [74]. With regards to complications, Vijayaraman *et al*. [64] found significantly higher procedural complications in patients undergoing BiVP versus LBBAP (7.5% vs 3.8%, *p <* 0.001), with higher rates of acute lead dislodgements, infections and pneumothoraces in the BiVP group. Diaz *et al*. [73] reported less than 1% risk of acute complications for both BiVP and LBBAP, however there was a trend towards higher long term complications in patients receiving BiVP, with higher rates of infection, lead dislodgement and phrenic nerve stimulation. The large multi-centre, registry-based, MELOS study analysed outcomes in patients undergoing LBBAP for both bradyarrhythmia’s and heart failure [74]. They reported acute and late complications in 11.7% of patients, with 8.3% of these complications related to the transseptal route. In particular, intraprocedural perforation into the LV cavity occurred in 3.7% of patients, as well as a very small number of patients developing coronary artery damage or spasm. Lead dislodgement rates were reported in 1.5% of cases, which remains lower than the reported rates of LV lead displacement in BiVP [15].

Despite HBP being associated with better electrical synchrony than LBBAP, these findings suggest LBBAP is an effective alternative pacing strategy to HBP. Like HBP, results suggest it is associated with favourable clinical and echocardiographic findings when compared to conventional BiVP. Due to more stable capture thresholds and favourable anatomy for lead placement, it may well be the preferred technique for CSP in the future. However larger randomised controlled trials with longer follow-up periods will be needed before it is recommended as a first line alternative to conventional biventricular CRT.

### 3.4 Conduction System CRT and Right Bundle-Branch Block

Despite the overwhelming evidence supporting the use of CRT in patients with LBBB, there remains limited evidence surrounding its use in non-LBBB morphologies. A meta-analysis of five randomised controlled trials in 2015 concluded that CRT did not improve mortality or heart failure hospitalisations in heart failure patients with broad QRS intervals and non-LBBB morphology [77]. However, since then evidence has emerged to suggest that conduction system CRT may be effective in this cohort of patients. Observational studies by Sharma *et al*. [78] and Vijayaraman *et al*. [79], have shown that CSP in patients with symptomatic HFrEF and right bundle branch block morphology is efficacious. They reported success rates of 88–95%, and both reported statistically significant reductions in QRS interval, improvements in LVEF and enhanced echocardiographic and clinical outcomes. More recently, Tan *et al. * [80] also reported favourable LV function and echocardiographic response with CSP versus BiVP in this cohort, along with a 78% reduction in-all cause mortality.

### 3.5 Conduction System Optimised Therapy

A new area of promise is the development of His-optimised CRT (HOT-CRT) and LBBA-optimised CRT (LOT-CRT). Many patients with advanced heart failure have distal and widespread delay in the conduction system or functional conduction block, resulting in delayed activation of the lateral part of the left ventricle. In practice, there is heterogeneity between LBBB patterns, and this can be difficult to determine from 12-lead ECGs. Electrophysiological studies have suggested around a third of patients with LBBB-pattern have intact purkinje activation, thereby suggesting that distal, intraventricular conduction defects are common [81]. It therefore may well be the case that patients with advanced heart failure have mixed disease, with proximal LBBB as well as intraventricular delay secondary to intrinsic myocardial disease.

In conduction system optimised therapy (CSP-OT), as well as a CSP lead there is also a coronary sinus lead providing electrical activation of the LV lateral wall. This results in ventricular fusion pacing and avoids late LV wall activation which may be seen in distal or intraventricular conduction delay. Early, small, non-matched observational studies have shown promising results for this therapy. Zweerink *et al*. [82] found a significantly reduced LV activation time in HOT-CRT patients versus patients with BiVP or HBP alone. Furthermore, Vijayaraman *et al*. [83] found HOT-CRT resulted in significant improvements in LV function, echocardiographic response, functional status and requirements for diuretic therapy. HOT-CRT also significantly reduced the QRS interval compared to HBP alone and achieved electrical synchronisation in patients with intraventricular conduction defects, in whom HBP had been unsuccessful.

Jastrzębski *et al*. [76] found that LOT-CRT was successful in 81% of patients. Similarly, they found that LOT-CRT significantly improved echocardiographic and functional outcomes and narrowed the QRS interval when compared to CSP alone. A recent multi-centre randomised controlled trial has proposed how CSP-OT could be utilised in clinical practice, by only inserting an additional LV pacing lead in patients with evidence of intraventricular conduction defects, mixed conduction disease or delayed lateral wall activation during CSP [84]. CSP-OT resulted in a significantly greater LVEF when compared to BiVP, however there was no significant difference in other outcomes, including heart failure hospitalisation, quality of life, QRS duration or functional status at 6 months follow-up.

### 3.6 Pace and Ablate 

For patients with AF and symptomatic heart failure refractory to medical therapy, ventricular pacing with AV node ablation is a well-recognised and effective treatment option. In patients receiving AV node ablations, studies have shown that BiVP when compared to RVP, is associated with improved LV function as well as reductions in heart failure hospitalisations and mortality [85, 86, 87]. Findings from the recent the Ablate and Pace for Atrial Fibrillation—cardiac resynchronization therapy (APAF-CRT) have suggested that BiVP with atrioventricular node ablation has favourable mortality outcomes when compared to medical therapy in patients with heart failure and symptomatic, permanent AF with normal QRS morphology [88]. There is now also growing evidence to suggest CSP may be a valid alternative pacing strategy to traditional BiVP in these patients, especially in those with normal QRS morphology as it allows intrinsic ventricular synchrony to be maintained [55, 89, 90, 91] (Table 2).

**Table 2.** **Studies comparing conduction system pacing to biventricular pacing in patients undergoing pacing and atrioventricular node ablation for persistent atrial fibrillation**.

Study	Study design	Operative period	Country	Follow-up	Patients (n)	QRS duration at follow-up	NYHA class at follow-up	LVEF at follow-up	Clinical outcomes
Vijayaraman *et al*. 2022 [89]	Retrospective observational study	2015–2020	United States of America (single centre)	Mean 27 ± 19 months	223 (110 CSP, 113 conventional pacing (either RVP or BiVP))	QRS duration increased in CSP (103 ± 25 ms to 124 ± 20 ms, *p * < 0.01) and conventional pacing groups (119 ± 32 ms to 162 ± 24 ms, *p * < 0.001). QRS duration significantly lower in CSP group (*p * < 0.01)	-	Improved in both CSP (46.5 ± 4.2% at baseline to 51.9 ± 11.2%, *p* = 0.02) and conventional pacing groups (36.4 ± 16.1% to 39.5 ± 16%, *p* = 0.04)	Lower rate of death or heart failure hospitalisation in the CSP group (48% vs 62%, Hazard Ratio 0.61 [0.42–0.89], *p * < 0.01)
Ivanovski *et al*. 2022 [55]	Retrospective observational study	2015–2022	Slovenia (single centre)	Mean 5 months	50 (13 BiVP, 25 HBP, 10 LBBAP)	QRS duration was significantly shorter in CSP than in BiVP (*p * < 0.001)	Improvement in NYHA class in BiVP group but not statistically significant (*p* = 0.096). Statistically significant improvement in NYHA class in LBBAP (*p* = 0.008) and HBP groups (*p * < 0.001)	No change in BiVP group (38% at baseline to 37%, *p* = 0.916) but significant improvements in LBBAP (28% at baseline to 40%, *p* = 0.041) and HBP groups (39% at baseline to 49%, *p* = 0.033)	Three patients (2 in BiVP group and 1 in HBP group) died during follow-up
Žižek *et al*. 2022 [90]	Retrospective observational study	2015–2020	Slovenia (single centre)	6 months	24 (12 BiVP, 12 HBP)	QRS duration remained unchanged in HBP group (91 ± 12 ms at baseline to 95 ± 15 ms, *p* = 0.281) and significantly prolonged in the BiVP group (from 98 ± 7 ms at baseline to 172 ± 13 ms, *p * < 0.0001)	Improvement in NYHA class in 75% of the HBP group and 50% of the BiVP group, however no significant differences between groups at follow-up (*p* = 0.212)	Improved in HBP group and decreased in BiVP group (7.2% vs –1.1%, *p* = 0.014)	-
Huang *et al*. 2022 [91]	Prospective randomised crossover trial	-	China, United States of America, United Kingdom (multi-centre)	18 months	50 (25 in each arm)	QRS duration was prolonged in both groups, but prolongation was significantly greater in the BiVP group (135.7 ± 16.6 ms vs 107.6 ± 12.5 ms, *p* = 0.001)	Greater improvement in NYHA class in HBP group (–1.3 [–1 to –1.6], *p* = 0.001) than BiVP group (–1.2 [–0.9 to –1.5], *p* = 0.001) but no statistical difference between the groups	Linear mixed-effects model revealed that HBP improved LVEF more than BiVP (*p* = 0.015)	Three hospitalisations occurred (1 during HBP and 2 during BiVP)

CSP, conduction system pacing; RVP, right ventricular pacing; BiVP, biventricular pacing; HBP, his-bundle pacing; LBBAP, left bundle branch area pacing; LVEF, left ventricular ejection fraction; NYHA, New York Heart Association.

Observational studies have shown that HBP, or alternatively LBBAP, with atrioventricular node ablation leads to significant improvements in both LV function and NYHA class in patients with refractory AF [92, 93]. The recent ALTERNATIVE-AF trial found HBP resulted in significantly greater LV function compared to BiVP in patients with HFrEF and persistent AF [91]. Both pacing modalities improved quality of life, NYHA functional status and BNP levels, with no significant differences between the groups. In the first study comparing HBP to LBBAP in patients undergoing ablation and pacing, Cai *et al*. [94] found that both pacing strategies resulted in significant improvements in NYHA classification and LVEF in patients with both reduced and preserved LV function. As well as a 100% implantation success rate, LBBAP was associated with a reduction in lead related complications compared to HBP and stable and lower thresholds at follow-up. It is well recognised that atrioventricular node ablations can result in increases in capture thresholds, and in this study significant increases in capture thresholds resulted in lead failure in 5.8% of HBP patients. As a result of this complication, ESC guidelines recommend adding RVP leads in this cohort [15].

## 4. Implantable Cardioverter Defibrillator Devices

Implantable cardioverter defibrillator (ICD) devices help prevent sudden cardiac death in patients at risk of ventricular arrhythmias. They are used for both primary and secondary prevention in high-risk patients and may be used in combination with other devices such as cardiac re-synchronisation therapy, as CRT-Defibrillator (CRT-D) devices. Like other transvenous devices, conventional ICD devices are associated with lead and pocket related complications. As well as this, despite reducing the risk of sudden cardiac death, data suggests high shock burden with ICD therapy is associated with higher mortality and heart failure hospitalisation rates [95]. Furthermore, inappropriate shocks are associated with anxiety, psychiatric co-morbidity and a reduced quality of life [96, 97]. This highlights the need for the careful selection of patients for ICD devices, after weighing up the potential risks and benefits.

### 4.1 Primary Prevention ICD Therapy in Patients with Non-Ischaemic Cardiomyopathy

**Fig. 2.**
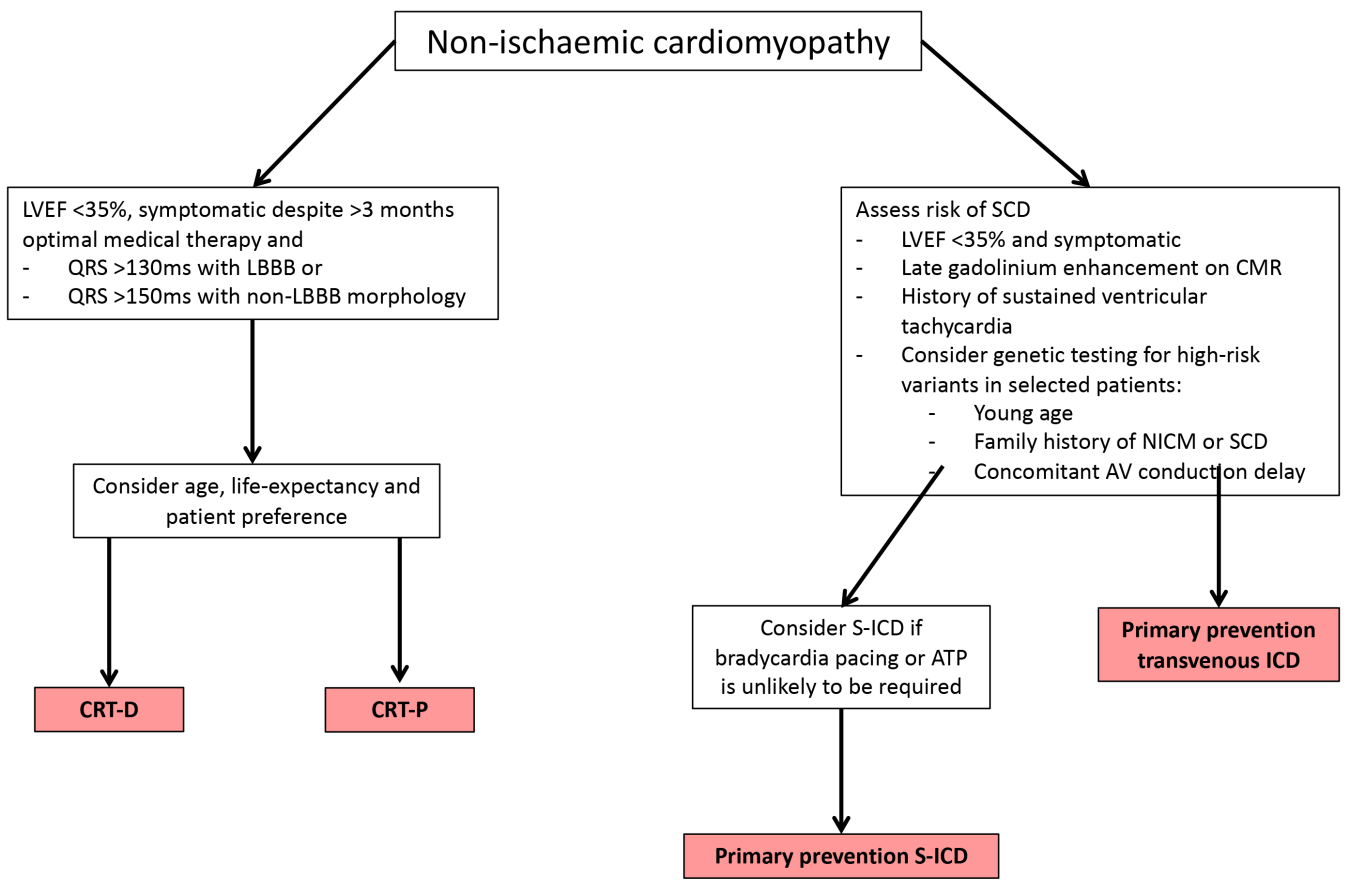
**Flow diagram to aid the selection of implantable defibrillator device in patients with non-ischaemic cardiomyopathy**. LVEF, left ventricular ejection fraction; LBBB, left bundle branch block; CRT-D, cardiac resynchronisation therapy defibrillator; CRT-P, cardiac resynchronisation therapy pacemaker; SCD, sudden cardiac death; CMR, cardiac magnetic resonance; NICM, non-ischaemic cardiomyopathy; ICD, implantable defibrillator cardioverter; S-ICD, subcutaneous implantable defibrillator cardioverter; ATP, anti-tachycardia pacing; AV, atrioventricular.

It is well accepted that ICDs are an effective therapy for primary prevention of sudden cardiac death in patients with ischaemic cardiomyopathy (ICM) [98]. The MADIT II trial found that ICD therapy significantly reduced all-cause mortality and arrhythmic death when compared to medical therapy in patients with coronary artery disease, ischaemic LV impairment and non-sustained ventricular tachycardia [99]. However, the role of ICD therapy for primary prevention in NICM is less well understood. Due to the heterogeneity of conditions causing NICM, recent ESC guidelines have primary prevention ICD therapy as a class IIa recommendation but only if patients fulfil certain criteria after diagnostic evaluation and risk stratification (Fig. 2) [98]. This is a change from conventional guidelines that have used LVEF cut-offs to risk stratify patients.

Several randomised controlled trials exist comparing the addition of ICD therapy to best medical therapy in patients with NICM [44, 100, 101, 102, 103]. Despite all trials highlighting a trend towards reduced mortality with ICD therapy, the difference was only statistically significant in the SCD-HeFT trial [103]. Several studies did, however, note significant reductions in sudden cardiac death with ICD therapy, and a trend towards favourable outcomes in younger patients. The results of these trials were combined in a meta-analysis by Masri *et al. * [104] who found the use of ICD therapy was associated with a significant 24% reduction in all-cause mortality and 60% reduction in sudden cardiac death. Since the DANISH trial in 2016 there have been no further randomised controlled trials comparing ICD to medical therapy in patients with NICM. However, Poole *et al*. [105] have since published data from the extended follow-up of patients in the SCD-HeFT trial and found that despite the original study reporting favourable mortality in patients with NICM, when this was extended to 10 years there was no difference in mortality between the groups. In all these existing studies, there have been very few asymptomatic or NYHA class IV patients, and therefore future randomised controlled trials should seek to clarify the benefit of ICD therapy in these cohorts.

Given the heterogeneity of aetiologies causing NICM, guidelines propose an individualised approach when making decisions about ICD therapy. Clinicians should consider aetiology, cardiac magnetic resonance (CMR) imaging findings, echocardiographic features, serological markers and genetic risk when considering the risk of ventricular arrhythmias and thereby the need for an ICD in patients [106]. In particular, CMR findings can be particularly useful in identifying areas of localised myocardial fibrosis that can provide an arrhythmic substrate, predisposing to ventricular arrhythmias and sudden cardiac death. Scarring can occur in both patients with and without severe LV impairment. A meta-analysis of 60 studies found that not only was late gadolinium enhancement (LGE) CMR effective at identifying scar tissue in NICM, but also that the presence of scar tissue was associated with a worse prognosis [107]. The presence of scar tissue predicted major ventricular arrhythmic events, all-cause mortality, cardiovascular mortality and hospitalisation with heart failure. Interestingly there was a statistically significant negative correlation between the effect sizes of all-cause mortality and age, suggesting that scar detection on LGE CMR is more significant in younger populations, who are less likely to die from alternative causes. These findings are supported by Gutman *et al*. [108], who found that primary prevention ICD therapy in NICM was only associated with a reduction in mortality if patients had LV scar tissue on CMR. Evidence suggests around 42% of patients with NICM have LGE on CMR, and that this along with LV function, are independent predictors of appropriate ICD therapy, sustained ventricular tachycardia, resuscitated cardiac arrest or sudden death [109]. Di Marco *et al*. [109] were then able to combine LGE status with LVEF to devise a risk predictive model for sudden death or ventricular arrhythmias, which was superior to LVEF cut-offs alone. The first randomised controlled trial using CMR LGE to guide ICD implantation in patients with NICM is recruiting patients (CMR-ICD trial) and will help provide further evidence on the benefits of CMR in this cohort [110].

As well as CMR, genetic variants can be used to risk-stratify patients with NICM. It is estimated that 20-30% of dilated cardiomyopathy (DCM) cases are familial and a genetic cause can be found in around 17% of patients [111]. Ebert *et al*. [112] found that 38% of patients with DCM undergoing ablation for ventricular tachycardia had pathogenic variants, most commonly Lid Margin Neovascularized Area (LMNA), Titin (TTN) and Phospholamban (PLN) variants. The presence of these variants was significantly associated with a reduced two-year ventricular tachycardia-free survival. Likewise, a 10-year analysis by Gigli *et al*. [113] identified desmosomal and LMNA gene variants as being the highest risk for ventricular arrhythmias or sudden cardiac death in patients with DCM. As well as this, several other pathogenic variants have been proposed including truncated mutations to FLNC, truncating variants of TTN and RBM20 variants. As a result, ESC guidelines advise genetic testing in younger patients with concomitant atrioventricular conduction block or in DCM patients presenting at an early age, as part of risk stratifying for consideration of ICD primary prevention therapy [98].

### 4.2 Choosing Between CRT-P and CRT-D 

In patients who have an indication for CRT, current ESC guidelines have a class IIa recommendation to consider adding a defibrillator function after considering individual risk assessments and involving the patient in shared decision making [15]. In patients with severe LV impairment, it is thought that adverse ventricular remodelling and diffuse interstitial fibrosis, can lead to self-organising criticality and electrical instability, predisposing to ventricular arrhythmias [114]. Despite new medical therapies including SGLT2i and sacubitril/valsartan being shown to be effective at reducing the risk of arrhythmias in these patients [115, 116], myocardial scar tissue does not resolve with CRT or medical therapy. These patients therefore continue to have a significant residual risk of ventricular arrhythmias, therefore suggesting a role for defibrillator devices. However, in the absence of ventricular remodelling, Deif *et al*. [117] have shown that CRT therapy may even be pro-arrhythmogenic through LV epicardial pacing. Along with this, as well as costs, CRT-D devices are associated with complications such as lead failure and inappropriate shocks. This therefore means that it is vitally important that CRT-D devices are only considered in patients who are likely to benefit from the therapy.

Previous meta-analyses have suggested CRT-D devices are associated with favourable mortality outcomes when compared to CRT pacemakers (CRT-P) [118]. A recent meta-analysis by Veres *et al*. [119] including 128,030 patients has added to this evidence and suggested it is superior to CRT-P in younger patients or in patients with ICM. They found that CRT-D was associated with a significant 20% reduction in all-cause mortality when compared to CRT-P. When excluding non-propensity matched studies this mortality reduction remained significant albeit slightly lower, which may be explained by CRT-D candidates being younger and having fewer co-morbidities than their CRT-P counterparts. This analysis, however, found no difference in mortality between the two groups in patients with non-ischaemic aetiology or in those over 75 years of age.

Whilst there appears to be strong evidence that CRT-D are associated with reduced all-cause mortality versus CRT-P in patients with ICM [120, 121, 122], results are inconsistent in patients with NICM. Like Veres *et al*. [119], a recent meta-analysis by Al-Sadawi *et al*. [123] also found no difference in mortality between CRT-D and CRT-P devices in patients with NICM. However, recent large-scale, retrospective observational studies with extended periods of follow up, have suggested that CRT-D are in-fact associated with reduced all-cause mortality in patients with NICM. Like the findings of previous meta-analyses there appears to be a relationship between CRT-D outcomes and age in patients with NICM. Gras *et al*. [124] found CRT-D were associated with reduced mortality compared to CRT-P in both ischaemic and non-ischaemic cohorts, however they observed no significant difference in the survival rates of patients over 75 years of age with NICM. Likewise, Farouq *et al*. [125] found that CRT-D were associated with a significantly reduced 5-year mortality rate, and although there was no linear association between age and mortality, they found the largest reduction in mortality was in patients below the age of 60. The observed favourable mortality outcomes in younger patients receiving CRT-D devices may be explained by the causes of death in these patients. Younger patients are more likely to suffer from sudden cardiac death due to ventricular arrhythmias rather than older patients, who are proportionately more likely to die from progression of their underlying heart failure and non-cardiac causes. This may explain why historical studies have seen the mortality benefit of CRT-D therapies attenuate over time [44, 120, 126]. Despite patients over the age of 75 years accounting for over half of patients with heart failure, they remain under-represented in many ICD clinical studies [99, 103, 127]. Furthermore, a meta-analysis by AlTurki *et al*. [128] found that patients over 75 years of age undergoing CRT had 74% reduced odds of having a defibrillator device compared to younger patients. In their analysis of medicare beneficiaries, Zeitler *et al*. [129] found CRT-D devices were associated with reduced mortality and heart failure hospitalisations in elderly patients with HFrEF when compared to ICDs alone. CRT-D devices however were associated with high complication rates (16.8% in patients over 75 years old) and mortality rates at 1 year (20.7% in 75–84 year olds and 24.8% in >85 year olds respectively). Current ESC guidelines only recommend defibrillator devices in patients who are expected to live over one year with a good functional status, and these high mortality and complication rates may explain the historical reluctance to implant devices in this cohort [130].

It should be noted that evidence on this topic is from observational studies and largely in non-propensity matched cohorts. The COMPANION trial is the only randomised controlled trial that exists to date where patients were randomised to CRT-D or CRT-P therapy, however this was designed to compare CRT to medical therapy and not the two devices [44]. Therefore, further randomised trials, such as the ongoing RESET-CRT trail [131], will be needed before firm conclusions can be drawn about the benefits of CRT-D devices over CRT-P.

As well as age and aetiology of heart failure, several other factors have been proposed as predictors of ventricular arrhythmias and therefore indications for CRT-D devices, including echocardiographic response to CRT and presence of myocardial scar tissue on CMR. A meta-analysis by Yuyun *et al*. [132] found that CRT response, as defined by echocardiographic criteria, was associated with a reduced risk of ventricular arrhythmias. They found that CRT-responders were significantly less likely to have appropriate ICD therapy due to ventricular arrhythmias than non-responders, and the pooled incidence of ventricular arrhythmias was significantly less in CRT super-responders. These findings are consistent with historical data that observed reduced ventricular arrhythmias in patients that responded to CRT [15]. Furthermore, there is now evidence to suggest that scar tissue detected on CMR could be used to guide CRT-D therapy decisions. Extension and heterogeneity of myocardial scar tissue has been shown to be an independent predictor of sudden cardiac death or need for ICD therapy, whilst the presence of scar tissue is associated with higher mortality, sudden cardiac death or sustained ventricular arrhythmias [133, 134, 135]. In patients with mid-wall fibrosis, Leyva *et al*. [134] found CRT-D was associated with a reduction in mortality and sudden cardiac death compared to CRT-P devices.

### 4.3 Subcutaneous ICD Devices

Long-term analyses of patients with ICD devices suggest as many as one in four will develop mechanical complications within 10 years of insertion [136]. Lead related complications include lead fracture and infection, and lead extraction is a high-risk procedure, with several serious potential complications including the need for emergency cardiac surgery [137]. Subcutaneous ICD (S-ICD) devices remain outside of the thoracic cavity, thereby eliminating the risk of lead-related complications and enabling safer device extraction. S-ICD devices, however, cannot be programmed to deliver pacing, which is significant given around 15% of patients with transvenous ICD devices develop an indication for downstream anti-bradycardia or resynchronisation pacing therapy after 5 years [138]. There are new technologies being developed which allow leadless pacemakers to be commanded by S-ICD devices, although this is currently experimental in humans and the subject of an ongoing randomised controlled trial [139]. Therefore, current guidelines suggest S-ICD devices should only be considered when bradycardia pacing, CRT or anti-tachycardia pacing is not required [98].

The only randomised controlled trial comparing outcomes between S-ICD and transvenous ICD devices, found there was no difference in device-related complications, mortality, major cardiac events or heart failure hospitalisation between the two cohorts at 49 months follow-up [140]. Device-related complications were higher in the transvenous ICD group, and the number of inappropriate shocks was higher in the S-ICD group, however neither achieved statistical significance. The most common cause for inappropriate shock in the transvenous ICD group was for supraventricular arrhythmias, whereas it was oversensing in the S-ICD group. As well as longer charging times, S-ICD devices are unable to deliver anti-tachycardia pacing, which may explain the higher number of inappropriate shocks in the S-ICD cohort. A recent systematic review and meta-analysis found that S-ICD devices are associated with a significantly reduced risk of lead-related complications, including cardiac perforation, pneumothoraces and lead failure or dislodgement when compared to transvenous devices [141]. There was a trend towards reduced device-related complications, which authors defined as complications requiring invasive intervention, in patients receiving S-ICD devices, but there were no significant differences in mortality, infection or inappropriate shock therapy between the groups.

Inappropriate shocks in transvenous ICD devices tend to result from supraventricular tachycardias, whereas they most commonly occur in S-ICD devices due to T-wave oversensing [141]. Whereas supraventricular tachycardias are easy to suppress with medications, oversensing in S-ICD devices is harder to correct. All patients undergoing S-ICD implantation have pre-implantation screening to ensure devices can distinguish T and Q waves, however there are now more sophisticated programming technologies to help navigate this risk. Boersma *et al*. [142] utilised dual zone tachycardia detection and observed a significant 36.4% reduction in inappropriate shocks in the first year of implantation. Similarly, Theuns *et al*. [143] used SMART pass methodology and saw a 68% reduction in inappropriate shocks. More recently the UNTOUCHED study reported an inappropriate shock rate of 3.1% at one year after using a novel vector selection method and device programming, which is comparable to transvenous devices [144]. In a real life, prospective cohort analyses of patients undergoing S-ICD implantation, the UNTOUCHED trial programming was associated with statistically significant reductions in inappropriate shocks, with no significant impact on appropriate shock delivery [144, 145].

Although there remains a relative lack of literature comparing S-ICD to transvenous ICD devices, published real life clinical data suggests that S-ICD is a safe alternative to traditional transvenous devices with reductions in lead related complications. Both ESC and American Heart Association guidelines reflect this and recommend their use in patients with complex venous anatomy, at high risk of infection or in those that do not require bradycardia pacing or anti-tachycardia pacing functions [98, 146]. More long-term randomised controlled trials comparing outcomes between subcutaneous and transvenous devices are likely to be needed, such as the anticipated findings of the ATLAS trial [147], before such devices have widespread use as a primary alternative.

## 5. Conclusions

Implantable devices remain at the forefront of the management of tachy- and brady-arrhythmias, and in recent years there have been several technological developments that aim to reduce their associated complications. CSP offers a more physiological alternative to RVAP in the management of brady-arrhythmias by reducing ventricular desynchrony. It is also an effective alternative to BiVP in patients receiving CRT, and can be combined with a LV lead to provide conduction system optimised pacing in patients with distal LBBB. Guidelines emphasise the importance of carefully selecting candidates for ICD devices. Literature now suggests that ICD’s are effective as primary prevention for sudden cardiac death in patients with NICM, as well as ICM. Furthermore, data now suggests CRT-D devices may be associated with favourable outcomes compared to CRT-P, especially in patients with ICM or younger patients. Novel leadless devices such as leadless pacemakers and S-ICD devices are in the early stages of development, but there is evidence to suggest that they are safe alternatives to transvenous devices and can reduce the risk of lead and pocket related complications. Moreover, new programming settings are helping to alleviate the risk of over sensing and inappropriate shocks in S-ICD devices.
